# Anti-HIV-1 Activity of Eight Monofloral Iranian Honey Types

**DOI:** 10.1371/journal.pone.0108195

**Published:** 2014-10-21

**Authors:** Mandana Behbahani

**Affiliations:** Department of Biotechnology, Faculty of Advanced Sciences and Technologies, University of Isfahan, Isfahan, Islamic Republic of Iran; Aligarh Muslim University, India

## Abstract

Monofloral Iranian honeys from eight floral sources were analyzed to determine their anti-HIV-1 activities as well as their effects on lymphocyte proliferation. The Peripheral Blood Mononuclear Cells (PBMCs) used in this study were prepared from five healthy volunteers who were seronegative for HIV, HCV, HBV and TB. The anti-HIV-1 activity of eight different honeys was performed by quantitative polymerase chain reaction (PCR) assay and high pure viral nucleic acid kit. The results demonstrated that monofloral honeys from *Petro selinum sativum*, *Nigella sativa*, *Citrus sinensis*, *Zataria multiflora*, *Citrus aurantium* and *Zizyphus mauritiana* flowers had potent anti-HIV-1 activity with half maximal effective concentration (EC_50_) values of 37.5, 88, 70, 88, 105 and 5 µg/ml respectively. However, monofloral Iranian honeys from *Astragalus gummifer* and *Chamaemelum nobile* flowers had weak anti-HIV-1 activity. The frequency and intensity of CD4 expression on PBMCs increased in the presence of all honey types. CD19 marker were also increased after the treatment with monofloral honeys from *Z.multiflora* and *N. sativa*. The anti-HIV-1 agent in monofloral honeys from *P.sativum*, *N. sativa*, *Z. multiflora* and *Z. mauritiana* flowers was detected by spectroscopic analysis as methylglyoxal. Time of drug addition studies demonstrated that the inhibitory effect of methylglyoxal is higher on the late stage of HIV-1 infection. The result demonstrated that methylglyoxal isolated from monofloral honey types is a good candidate for preclinical evaluation of anti-HIV-1 therapies.

## Introduction

The human immunodeficiency virus type 1 (HIV-1) is one of the most infectious agents causing disease and death through depletion of CD4 lymphocytes and immune-suppression [1]. Current anti HIV drugs have a lot of disadvantages including resistance, toxicity and limited availability. Many studies have been carried out worldwide to develop drugs that inhibit diverse steps of viral replication and improve immunologic parameters [2], [3]. Natural products have been considered as potential anti-HIV drugs [4]. Some important secondary metabolites that are obtained from these products including alkaloids, flavonoids, sulphated polysaccharides, coumarines and triterpenes [5], [6], have been described to inhibit different steps of viral replication such as reverse transcription process, virus entry and the integrase or protease activities [7], [8]. Screening anti-HIV compounds from natural products may be one of the effective ways to discover new drugs. Honey is a tasty natural product which has been consumed for its high nutritive value and its role in human health. The value of honey is determined by its chemical and physical characteristics [9]. A diversity of secondary metabolites, minerals, proteins, free amino acids, enzymes, and vitamins have been obtained from this product [10]. Several biological activities of honey such as antioxidant and anti-bacterial have been previously investigated [11]. Recently, methylglyoxal has been documented as a potent antibacterial agent in manuka honey. However there is no report about anti-HIV-1 activity of methylglyoxal. Therefore, we became eager to assess Iranian species with respect to their anti-HIV-1 activity [12]. The anti-HIV potency of insect products derived from honey were also reported [13]. Al-Waili *et al.* demonstrated that natural honey decreased prostaglandins level and improved hematological and biochemical tests in a patient with AIDS [14]. Ethiopian multiflora honey were reported to treat resistant Candida strains in AIDS patients [15]. The composition of honey is dependent on its floral sources and environmental factors. The variation of honey composition may be responsible for different biological activities [16]. Honey production in Iran has very long traditions and it is famous for its quality. However, little information is available about biological activity of honey from different floral sources in Iran. Therefore, in the present study, the anti-HIV1 effects of eight different monofloral honeys and their effects on expression of lymphocyte activation markers have been studied.

## Materials and Methods

### Ethics statement

This study was approved by the Ethical Committee in Isfahan University, Iran. All participants were provided written informed consent to participate in the study. The written consents of participation were approved by Isfahan University.

### Honey samples

Monofloral honeys from eight floral sources were obtained from three different centers of Iran including Jihad-agricultural organization of Mazandaran, Fars Agricultural Organization and National Center of Agriculture Research of Isfahan. The eight different floral sources were *Petro selinum sativum*, *Nigella sativa*, *Citrus sinensis*, *Zataria mmultiflora*, *Citrus aurantium*, *Zizyphus mauritiana*, *Astragalus gummifer and Chamaemelum nobile*. These samples were certified as monofloral by the National Center of Agricultural Research of Isfahan, Iran. Fresh honey samples weighing 50 g were packed and sealed in amber glass bottles and stored at 4°C in the dark.

### Extraction and isolation of compounds

Four monofloral Iranian honeys isolated from *P. sativum*, *N. sativa*, *Z. multiflora* and *Z. mauritiana* (5 g) were separately dissolved in 5 ml methanol and fractionated separately using silica-gel column chromatography. The methanol extracts were separately eluted with Hexane: Acetone: Methanol (10∶0∶0 – 0∶0∶10, v/v/v). The following fractions have been obtained from these monofloral Iranian honeys:

Fractions 1–24 from monofloral Iranian honeys isolated from *Z. mauritiana* (0.31, 0.22, 0.17, 0.21, 0.15, 0.13, 0.24, 0.19, 0.14, 0.24, 0.22, 0.35, 0.21, 0.14, 0.12, 0.11, 0.21, 0.22, 0.29, 0.14, 0.11, 0.24, 0.2, 0.21 g), *P. sativum* (0.21, 0.28, 0.21, 0.2, 0.2, 0.12, 0.17, 0.11, 0.19, 0.19, 0.13, 0.17, 0.18, 0.27, 0.21, 0.1, 0.11, 0.12, 0.11, 0.22, 0.24, 0.31, 0.33, 0.48 g), *N. sativa* (0.2, 0.10, 0.24, 0.26, 0.32, 0.19, 0.18, 0.14, 0.2, 0.19, 0.16, 0.2, 0.22, 0.19, 0.17, 0.21, 0.2, 0.22, 0.23, 0.21, 0.23, 0.15, 0.23, 0.16 g) and *Z. multiflora* (0.3, 0.31, 0.25, 0.17, 0.19, 0.17, 0.21, 0.2, 0.11, 0.17, 0.1, 0.21, 0.18, 0.17, 0.21, 0.1, 0.23, 0.2, 0.21, 0.15, 0.22, 0.23, 0.2, 0.22 g) were obtained. The anti-HIV-1 activity of all of the fractions was tested and the most active fractions were selected. Fractions 10, 12, 14 and 19 respectively from *N. sativa*, *Z. mauritiana*, *P. sativum* and *Z. multiflora* have been demonstrated to have potent anti-HIV-1 activity and analyzed by NMR. All solvents and chemicals used for experimental purposes were purchased from Merck (Germany).

### Cells and viruses

Peripheral Blood Mononuclear Cells (PBMCs) from five healthy volunteers who were seronegative for HIV, HCV, HBV and TB (approved by blood transfusion, Alzahra hospital, Isfahan, Iran) were separated on density gradient centrifugation (lymphodex). This study was approved by the Ethical Committee in Isfahan University, Iran. The PBMCs were grown in RPMI medium supplemented with 10% (v/v) heat inactivated Fetal Calf Serum (FCS; Gibco-BRL, Grand Island, NY, USA), 100 U/ml penicillin, 100 mg/ml streptomycin, 2 mM glutamine and 1 mM Na-pyruvate and activated with 5 µg/ml phytohemagglutinin (PHA) and IL-2. All reagents were purchased from Gibco. Purified HIV-1 subtype A was obtained from Alzahra Hospital, Isfahan, Iran. Aliquots of 6×10^5^ cells per well were infected with HIV-1. Virus titers were measured by 50% cell culture infectious dose (TCID50) by end point dilution. The virus stocks with titers as high as 6×10^4^ infectious units/ml were obtained. The viruses were stored in medium containing dimethyl sulfoxide (DMSO) in liquid nitrogen (−70°C).

### Antiviral activity

Anti-HIV-1 activity of eight monofloral honey types and their fractions was studied via the HIV-1 P24 antigen kit. This kit is used to measure the amounts of HIV-1 Gag P24 antigen in cell culture medium. The PBMCs were grown on microtitre tissue culture plates. The cells were infected with 0.5 Multiplicity of Infection (MOI) HIV-1 and supplemented with different concentrations of monofloral honey types (100, 50, 10 and 5 µg/ml) and incubated at 37°C for 12 hours. Infected cells were washed and overlaid with medium. DMSO (0.1%) and different concentrations of Zidovudine (AZT) (5, 2.5, 1, 0.1and 0.01 µg/ml) were used as negative and positive controls respectively. After 72 hours of incubation, the overlay medium was used to detect and quantify HIV-1. At the end, the supernatant was transferred to the 96-well plate coated with HIV-1 P24 antigen (bioMérieux, France). The protocol was followed as described by the manufacturer, with absorbance measured at 450 nm. The antiviral activity of active fractions was further examined by different concentrations (5, 2.5, 1, 0.1and 0.01 µg/ml).

### Human PBMC cytotoxicity assay

The PBMC cytotoxicity was estimated in the presence of eight monofloral honey types and active fractions using MTT assay [17]. Dilutions at concentrations of 10, 100, 1000 and 2000 µg/ml were added to PBMC (6×10^5^ cell per well) and incubated at 37°C and 5% CO2. After 3 days of culture, 20 µl of MTT (0.5 mg/ml) was added to each well and incubated for 2 hours in the same condition. Then, 50 µl of PrOH/HCl/TX (0.04 MHCL/2 iPrOH/10% triton ×100) was separately added to each well and incubated for 6 hours. The optical density of cells was measured at 570 nm by micro plate spectrophotometer (Awareness Technology Inc, Stat fax 2100). Each concentration was tested three times. DMSO was used as the negative control.

### Analysis of CD4, CD3, CD45 and CD19 expression by flow-cytometry

The percentage of CD4, CD3, CD45 and CD19 T-lymphocyte subsets and their expression intensities on PBMCs in the presence of eight monofloral honeys and active fractions were evaluated by flow-cytometry. 5×10^6^ PBMCs were cultured in 24-well plates. After 72 h of incubation at 37°C, the cells were washed with PBS. Cells were separately incubated with saturating concentrations of PE anti human CD4, RPE CY5 anti human CD3, FITC anti human CD45 and FITC anti human CD19 monoclonal antibodies (Cyto Matin Gene, IRAN) for 20 min at 4°C. Lymphocytes were gated based on their forward and side scatter properties. At least, 10000 events were acquired for each sample. Each incubation was followed by two washing steps. The flow-cytometer was a FAC Scan from Becton Dickinson. Data acquisition were achieved using BD Cell Quest software.

### Quantitative real-time PCR assay for HIV-1

For real-time PCR, 200 µl of cell culture supernatant from each treated or untreated groups (virus control) were collected. RNA was extracted from 200 µl of each group of cells by High Pure Viral Nucleic acid kit (Roche Diagnostics, Meylan, France) according to a standard protocol. The quantification of HIV-1 RNA copies was carried out by real-time PCR assay. As previously described, forward primer NEC152 (5′-CCTCAATAAAGCTTGCCTTGA-3′), reverse primer NEC131 (5′-GGCGCCACTGCTAGAGATTTT-3′) and the dually labeled NEC-LTR probe(5′-6-carboxyfluorescein-AGTGTGTGCCCGTCTGTTRTKTGACT-6-carboxytetramethylrhodamine-3′) in the long terminal repeat gene were used [18]. Primers and probes were synthesized by Metabion Co. (Germany). The master mix contained 1× RNA master hybridization buffer, including the Tth DNA polymerase and deoxynucleoside triphosphate mix containing dATP, dCTP, dGTP, dTTP and dUTP, 2.5 Mn (OAC) 2, and 0.3 µM concentrations of each primer and probe. The quantitative real-time PCR conditions were as follows: initial reverse transcription at 61°C for 30 min, denaturation at 95°C for 30 s, and 45 cycles of denaturation at 95°C for 1 s, annealing at 55°C for 15 s and elongation at 65°C for 1 min with a ramp of 5°C/s (with fluorescence acquisition at the end of each elongation stage). The positive control was consisted of culture supernatant of the HIV-1. As a control for cross-contamination, a sample of distilled water was subjected to the RNA extraction procedure, and the resulting extract was amplified in duplicate [19].

### Time of addition study

The time-of-addition experiment was carried out as described previously by Tao *et al.*, 2007 [20]. In brief, PBMCs, 2×10^5^ per well, were seeded into 24-well culture plates and incubated for 24 h. The cultures were treated with 0.5 MOI HIV-1 for 1 hour at 4°C to allow HIV-1 to attach the cells without fusion, and then washed three times with medium to remove unbound viruses. Active fraction and AZT at concentration of 5 µg/ml were added at different time points (0, 2, 4, 6, 8, 12, 16, 24, and 32 hours post infection). After incubation at 37°C for 72 h, the reduction in the virus titer in the cell culture supernatants was measured by quantitative real-time PCR assay and infection cultures containing the extracts were compared with that of the control cultures for each treatment.

### Statistical analysis

The data represent the mean ± SD from three independent experiments. The half-maximal cytotoxic concentration (CC50) and EC_50_ values were estimated using regression line analysis in Microsoft Excel 2007. A selectivity index (SI) was used to determine the effectiveness of anti-HIV agents. SI was calculated as the ratio of CC50 to EC_50_. The Analysis of Variance (ANOVA) was performed and a P value of <0.05 was used as the measure of statistical significance between samples.

## Results

### Spectroscopic analysis

**Figure 1:**
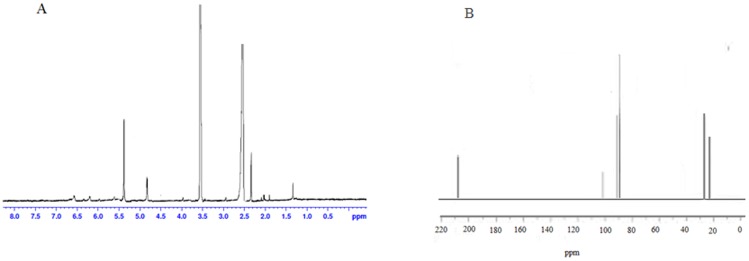
H NMR (A) and 13 C NMR (B) spectrum of methylglyoxal. The NMR data present a mixture of mono- and dihydrate forms of methylglyoxal.

Among the tested fractions, fractions 10, 12, 14 and 19 from *N. sativa*, *Z. mauritiana*, *P. sativum* and *Z. multiflora* were more active to reduce the viral titer of HIV-1 DNA (data not shown). The NMR data of these fractions were compared with similar studies on methylglyoxal [21]. These fractions were determined by NMR as mono and dehydrated forms of methylglyoxal (Fig. 1).

#### Methylglyoxal

**^1^H NMR (400 MHz, DMSO)** δ: 2.33 (s, 3H, CH3), 1.35 (s, 3H, CH3), 5.4 (s,1H, CH), 4.82 (s,1H, CH). **^13^C NMR (300 MHz, DMSO)** δ: 24.1,90.1, 187.1, 21.5, 97.1, 108.7 **IR (KBr) _νmax_:** 3089, 2936, 2870, 1719, 1710.

The percentage of the methylglyoxal fractions in mono-floral honey types from *N. sativa*, *Z. mauritiana*, *P. sativum* and *Z. multiflora* were 3.8, 7, 5.4 and 4.2 respectively.

### Anti-HIV-1 activity

**Figure 2:**
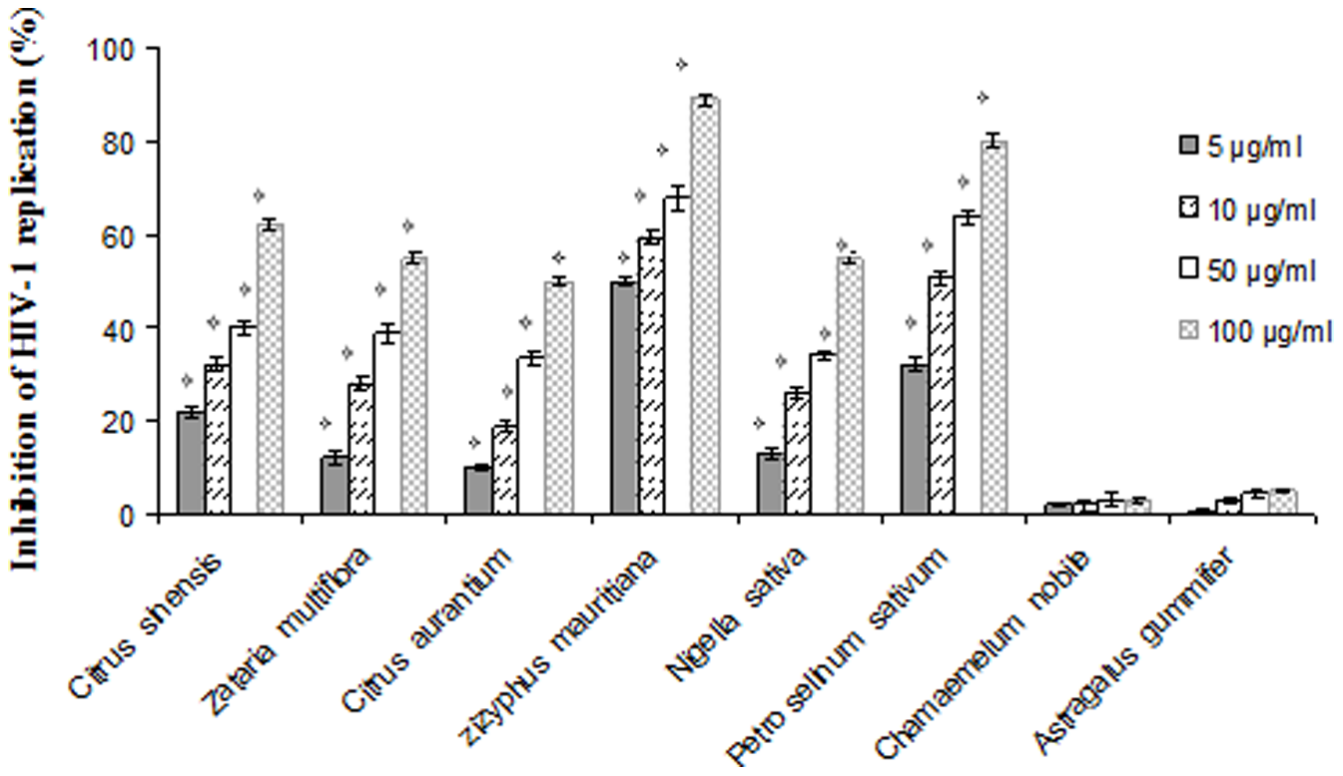
Effect of eight monofloral Iranian honeys at different concentrations on HIV-1 replication. The EC_50_ of each extract was calculated using regression line. Each bar represents the mean±SD of three independent experiments. The stars indicate that the data are significantly different (p<0.05) from the untreated control.

**Figure 3:**
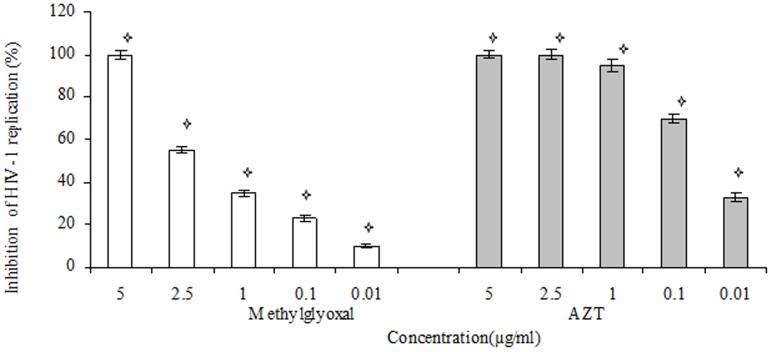
Effect of Methylglyoxal and AZT on HIV-1 replication. The EC_50_ of each extract was calculated using regression line. Each bar represents the mean±SD of three independent experiments. The stars indicate that the data are significantly different (p<0.05) from the untreated control.

Results showed that six types of honey originated from *C. sinensis*, *Z. multiflora*, *C. aurantium. Z. mauritiana*, *N. sativa* and *P. sativum* flowers inhibited viral replication in a dose dependent manner. Two honey types originated from *C. nobile* and *A. gummifer* flowers didn't have any antiviral effect. The effective concentrations of mono-floral honey types from *P. sativum*, *N. sativa*, *C. sinensis*, *Z. mmultiflora*, *C. aurantium* and *Z. mauritiana* flowers to reduce virus titers by 50% were 37.5, 88, 70, 88, 105 and 5 µg/ml, respectively. Also the calculated SI for mono-floral honeys originated from *C. sinensis*, *Z. multiflora*, *C. aurantium*, *Z. mauritiana*, *N. sativa* and *P. sativum* flowers were 21.4, 22.72, 22.7, 350, 17.04 and 40 respectively (Fig. 2). Methylglyoxal showed anti-HIV-1 activity more than eight honey types with EC_50_ and SI values of 2.2 and 590 µg/ml (Fig. 3).

### Analysis of Human PBMC cytotoxicity assay

**Figure 4:**
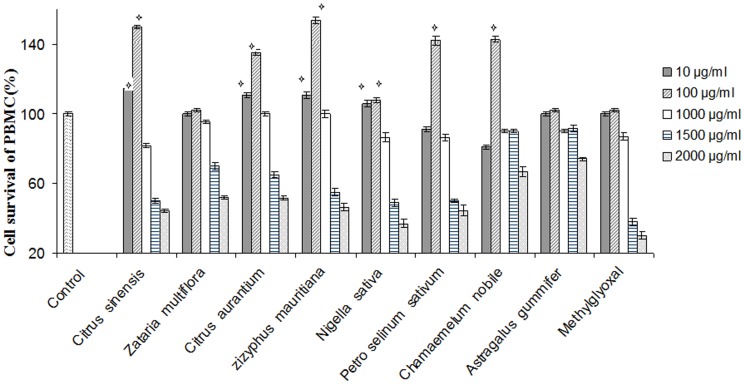
Effect of eight monofloral Iranian honeys and methylglyoxal at different concentrations on PBMCs proliferation assay. The cells treated with 0.1% DMSO were used as control. The stars indicate that the data are significantly different (p<0.05) from the untreated control.

Cellular toxicity of eight monofloral honey types on PBMCs is shown in Figure 4. The results showed that all honey types increased PBMCs number in a dose dependent manner. All samples increased PBMCs number up to 1.5 folds. The maximum proliferative effects of all samples were achieved at a concentration of 100 µg/ml. All extracts have a cytotoxic effect at higher concentrations. The result showed that CC50 values of all types were more than 1500 µg/ml. The CC50 values of eight mono-floral honeys were obtained at concentrations of 1500, 2000, 2000, 1750, 1500, 1500, >2000 and >2000 µg/ml which were originated from *C. sinensis*, *Z. multiflora*, *C. aurantium*, *Z. mauritiana*, *N. sativa*, *P. sativum*, *C. nobile* and *A. gummifer* flowers respectively. Methylglyoxal didn't have any effect on lymphocyte proliferation up to 100 µg/ml and had cytotoxic effect at higher concentrations. The CC50 value of methylglyoxal was obtained at 1300 µg/ml.

### Effect of honey types on expression of CD4, CD3, CD45 and CD19

The effects of eight monofloral honey types on frequency and the average mean fluorescent intensity (MFI) of CD4+ T cells in PBMCs are summarized in Table 1. The results showed that all honey types at concentration of 100 µg/ml increased frequency of CD4+ T cells in PBMCs. The average MFI value of the entire cell population in cells treated with all eight types were also increased up to a ratio of two folds compared to control. The results also showed that all studied monofloral Iranian honeys didn't have any effect on the intensities of CD3 and CD45 markers. The average MFI value and frequency of CD+19 lymphocytes were increased for the cells treated with *Z. mmultiflora* and *N. sativa* compared to control (Table 1). Methylglyoxal din't have any effect on the frequency and intensity of CD4, CD3, CD19 and CD45 markers.

**Table 1:** The effects of eight monofloral honey types on frequency and the average mean fluorescent intensity (MFI) of CD4, CD3, CD45 and CD19 in PBMCs.

*Monofloral Honey*	CD4		CD3		CD45		CD19	
	Frequency	MFI	Frequency	MFI	Frequency	MFI	Frequency	MFI
***Control***	20.23	18.10	81.35	265.94	88.16	207.30	3.67	14.9
***Citrus sinensis***	42.68	39.64	77.04	269.94	81.3	189.85	3.25	13.3
***Zataria mmultiflora***	42.27	29.90	85	264	87.98	173.40	4.22	24.72
***Citrus aurantium***	40.1	36.5	71.1	254. 45	80.41	162.15	4.1	14.85
***Zizyphus mauritiana***	40.54	40.39	79.36	300.54	87	202	3.6	12.1
***Nigella sativa***	44.75	46.79	82	255.1	89.10	208.15	3.5	27.78
***Petro selinum sativum***	42.15	41.37	82.4	250.02	83.1	210.4	3.4	11.2
***Chaemelum nobile***	38.5	36.8	80	251	83.5	208.4	3.41	14.1
***Astragalus qummifer***	41.2	40.5	85	244	82.16	201.5	3.6	13.8
***Methylglyoxal***	20.1	19.0	82.14	259.18	87.10	202.34	3.79	15.1

### Time of addition study

**Figure 5:**
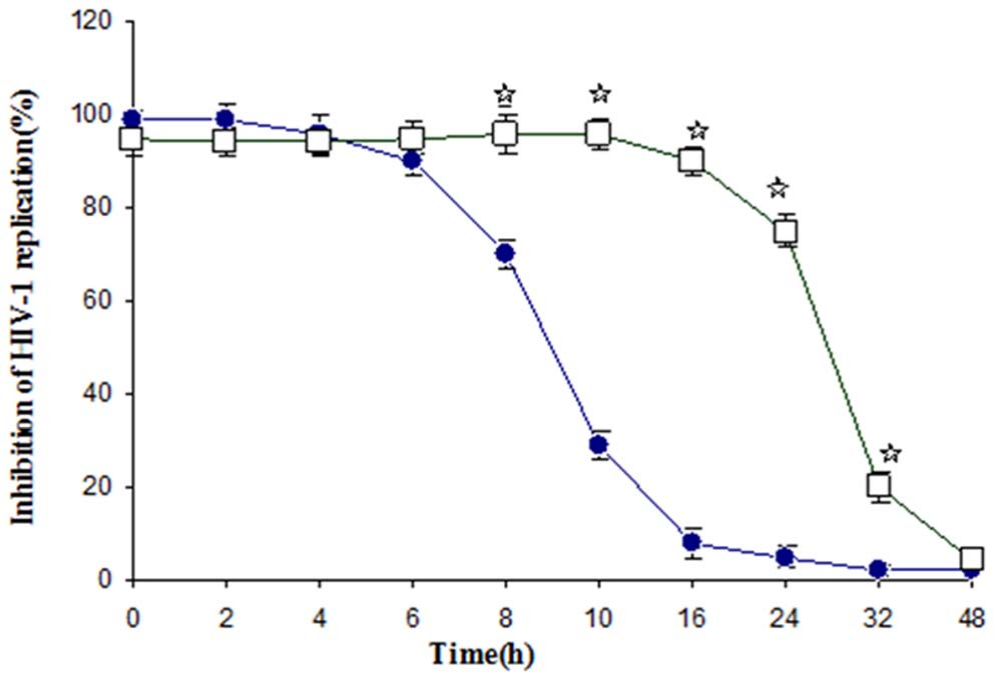
Time of addition effect of methylglyoxal (□) and AZT (•) at concentration of 5 µg/ml on HIV-1 replication. The extracts were added 0, 2, 4, 6, 8, 10, 16, 24, 32, 48 hours after virus infection. Each value is the result of mean ± SD of three independent experiments. The stars indicate that the data are significantly different (p<0.05) from the untreated control.

A time-of-addition experiment was performed by measuring the HIV-1 RNA yields in the infected culture supernatants, by means of a real-time PCR assay. The inhibition of HIV-1 replication in cells treated with methylglyoxal was achieved when the extracts were added between 0 and 24 hours post-HIV infection. The amount of viral DNA in methylglyoxal treated cells dramatically decreased when it was added at 32 and 48 hours post-HIV infection. As a control, AZT, a reverse transcriptase inhibitor, showed higher anti-HIV-1 activity when added between 0 and 6 hours post-HIV infection (Fig. 5). These data suggest that methylglyoxal inhibits HIV-1 infection by targeting viral life cycle after reverse transcription activity.

## Discussion

These studies suggest that there is a striking correlation between anti-HIV-1 activity of Iranian honeys and floral sources. In the present study, mono-floral honeys originated from *P.sativum*, *N. sativa*, *C. sinensis*, *Z. mmultiflora*, *C. aurantium and Z. mauritiana* showed potential anti- HIV-l activity while mono-floral honeys originated from *A. gummifer* and *C. nobile* showed lower anti-HIV activity. These results can be attributed to the differences in the secondary compound profile which are dependent largely on the floral source of the honey. Some reports have demonstrated that the biological activities of the most important monofloral honeys are related to the phytochemical composition and different geographical origins [22], [16]. Previously a correlation between the antioxidant activity of honey and proline content is reported. It suggests that honey amino acid content can play a role in its antioxidant activity [23]. The present results confirm the occurrence of methylglyoxal in monofloral Iranian honeys as a potent anti-HIV1 agent. The content of the methylglyoxal fraction in mono-floral honey from *Z. multiflora* was significantly more than mono-floral honey types from *N. sativa*, *Z. mauritiana* and *P. sativum*. Therefore, there is a relationship between anti-HIV-1 activity of mono-floral honey types and methylglyoxal level.

The anti-HIV-1 activity of methylglyoxal is distinguished here on the late stage of HIV infection and after reverse transcription. So, methylglyoxal may block assembly of new HIV virions and maturation of the virus. The assembly of new HIV virions is the ultimate step of the viral cycle which begins at the plasma membrane of the host cell [24]. Some commercial drugs such as saquinavir, indinavir, ritonavir, nelfinavir, amprenavir, lopinavir, atazanavir and fosamprenavir have been reported to inhibit protease activity and maturation of the new virus [25]. However, this is the first time that an alpha-keto aldehyde has been shown to impact the late stage of virus replication. It may provide the basis for the design of inhibitors that target the late stage of the viral life cycle. The results also demonstrated that frequency and intensity of CD4 expression in PBMC increased in the presence of all honey types. However methylglyoxal didn't show any effect on the lymphocyte proliferation. According to the previous result, the honey products such as propolis and prophylaxis can increase interferon-gamma (IF gamma) serum level and ratio of CD4+: CD8+ T-cell [23]. In the present study, anti-HIV-1 activity of methylglyoxal was significantly more than eight honey types, giving the conclusion that methylglyoxal is potent constituent in honeys to suppress HIV-1 activity. Our data demonstrated that Iranian honey types with high concentration of methylglyoxal might be good candidates for preclinical evaluation of anti-HIV-1 therapies.
